# Enhanced Dielectric and Mechanical Properties of Ternary Composites via Plasticizer-Induced Dense Interfaces

**DOI:** 10.3390/ma11071111

**Published:** 2018-06-29

**Authors:** Yefeng Feng, Cheng Peng, Yandong Li, Jianbing Hu

**Affiliations:** School of Materials Science and Engineering, Yangtze Normal University, Chongqing 408100, China; feng_ye_feng@126.com (Y.F.); andyydlee@gmail.com (Y.L.); hjb2008@163.com (J.H.)

**Keywords:** dielectric, mechanical, nanocomposite, plasticizer, interface

## Abstract

High overall performance, including high dielectric constant, low loss, high breakdown strength, fine flexibility, and strong tensile properties, is difficult to achieve simultaneously in polymer nanocomposites. In our prior work, we modified the surfaces of alpha-SiC nanoparticles and chemically cross-linked the polymeric matrix to simultaneously promote the dielectric and mechanical properties of composites. In this work, a novel strategy of high-temperature plastification towards a polymeric matrix has been proposed to fabricate ternary nanocomposites with balanced dielectric and mechanical characteristics by the solution cast method in order to reduce costs and simplify steps during large-scale preparation. Poly(vinylidene fluoride-chlorotrifluoroethylene) with inner double bonds as matrix, unfunctionalized alpha-SiC nanoparticles (NPs) as filler, and dibutyl phthalate (DBP) as plasticizer were employed. By introducing DBP and high-temperature treatment, the dispersion of NPs and the degree of compactness of the interface regions were both improved due to the reduced cohesion of the fluoropolymer, resulting in an increase in the dielectric constant (by 30%) and breakdown strength (by 57%) as well as the lowering of loss (by 30%) and conductivity (by 16%) in nanocomposites. Moreover, high-temperature plastification contributed to the promotion of flexible and tensile properties. This work might open the door to large-scale fabrication of nanocomposite dielectrics with high overall properties through the cooperation of the plasticizer and high temperature.

## 1. Introduction

High-dielectric-constant (high-k) materials possessing high flexibility and easy processing have attracted attention in the field of state-of-the-art electronic devices such as electroactive drivers and embedded capacitors [1,2,3]. The merits of polymer dielectrics are high electric breakdown strength (300–500 MV m^−1^) and easy processability, while their shortcomings are low dielectric constant (2–3) and thermal resistance (150–250 °C) [4], attributed to their covalent bonds. On the contrary, inorganic dielectrics with ionic bonds (such as ferroelectric barium titanate ceramic) have the virtues of rather high dielectric constant (1000–3000) and heat stability (800–1200 °C), but the weaknesses of low breakdown strength (10–50 MV m^−1^) and complicated processability [4]. Hence, blending can lead to a combination of the advantages of both composites.

So far, high-k composites made from polymer and ferroelectric ceramic have been widely studied [5,6,7,8,9,10,11]. For achieving a sufficiently high dielectric constant (circa (ca.) 100 [12]) in composites, researchers have to incorporate ferroelectric ceramic at a rather high volume content based on binary series and parallel dielectric models [13,14]. Unfortunately, uneven dispersions and aggregation of the ceramic in polymer would be induced, resulting in the reduced flexibility and mechanical robustness of the composites [15,16,17,18,19,20,21]. Furthermore, if nanosized ceramic filler with ultrahigh surface energy is introduced at a very high concentration, the mechanical characteristics would deteriorate.

Apart from ferroelectric ceramics, conductive and semi-conductive materials with lower band gaps have been added into polymers to construct high-k composites as well [22,23,24,25], based on interface polarization [26,27,28,29,30,31] and electric percolation threshold [32,33,34,35,36]. Under an applied electric field, movable charges would gather in interface zones between two materials with different intrinsic dielectric constants or conducting properties. When the electric conducting network across the whole composite is about to be formed among conducting particles (the particle content is rather close to a certain critical value), the quantity of gathered charges would be increased with an increase of the particle content or the applied field. Approaching that critical filler content can lead to an increase in the dielectric constant of the composites. To avoid the “insulator-conductor” transition, conducting particles are often introduced at a lower concentration. That can lead to a mild decline of flexibility in composites [37,38,39,40,41,42]. Unfortunately, these composites would lose their dielectric nature under higher fields due to elevated dielectric loss from interface leakage conduction [43]. Worse still, the loss at lower frequency ascribed to interface polarization relaxation might be too high to be accepted even in low field applications.

Although high dielectric performance has been achieved through blending polymer dielectrics and inorganic dielectrics, simultaneously obtaining high dielectric and mechanical properties based on the blend strategy is still a tough nut to crack. Nowadays, favorable mechanical characteristics of high-k composites are preferred [44,45,46]. In our previous work [47], both the dielectric and the mechanical properties of alpha-SiC/fluoropolymer composites were balanced by the surface modification of SiC and chemical cross-linking of the polymer. In order to further lower the costs and simplify the operation to accommodate large-scale fabrication of composites, in the present work we show another, easier strategy to simultaneously increase the dielectric and the mechanical properties in novel ternary nanocomposites through combining matrix plastification with high-temperature treatment. Firstly, neat alpha-SiC nanoparticles (NPs) were selected as filler to construct composites due to their wide band gap (ca. 3.09 eV), high breakdown strength (ca. 3200 kV cm^−1^), and moderate permittivity (ca. 10) [48]. Thereby, the shortcomings of high leakage conducting loss, low electric breakdown, and quick percolation in conductor/polymer composites would be avoided. Moreover, soft poly(vinylidene fluoride-chlorotrifluoroethylene) with inner double bonds (P(VDF-CTFE-DB)) in amorphous phase was employed as the matrix (without cross-linking). The reasons for choosing it were its high interface interaction with SiC NPs [47] (for high-k of composites), close intrinsic dielectric constant (ca. 12 at 100 Hz [47]) to NPs (for even allocation of electric field), and low Young’s modulus (ca. 13 MPa [49]) (for further plastification). Lastly, aiming at improving the compactness degree of interface zones and dispersion of NPs in composites, an easily available small molecule plasticizer namely dibutyl phthalate (DBP) was added into composites with SiC and P(VDF-CTFE-DB) to soften P(VDF-CTFE-DB) with the aid of high temperature. Note that the “compactness degree” is defined as the reciprocal of the air volume content inside the entire composite and was measured via a weight cup method [50]. This promoted a better dispersion of NPs in the matrix and reduced the number of air voids in interface regions thanks to high-temperature plastification of the matrix. Dense interface zones between NPs and polymer were responsible for the improved dielectric characteristics, including high permittivity (43 at 1 kHz) and breakdown strength (371 kV cm^−1^), and low loss and conductivity gained in ternary nanocomposites bearing DBP. Meanwhile, improved mechanical properties such as high flexibility were ascribed to the compacted interface zones as well. This work might open the door to a cheaper and faster large-scale fabrication of composite dielectrics with high comprehensive properties.

## 2. Materials and Methods

Silicon carbide nanoparticles in alpha crystalline form (α-SiC NPs) were purchased from Shanghai Xiangtian Nanomaterials Co., Ltd., Shanghai, China. α-SiC NPs (with real density of ca. 3.2 g cm^−3^) were washed with absolute ethyl alcohol four times to remove impurities, followed by thorough drying at 200 °C for 8 h before utilization. Poly(vinylidene fluoride-chlorotrifluoroethylene) (P(VDF-CTFE)), with CTFE units of 20 mol % as well as weight-average molecular weight of 120,000, was bought from Solvay (Shanghai, China). Dibutyl phthalate (DBP, 99%, AR grade, density ca. 1.04 g cm^−3^, boiling point 340 °C), absolute ethanol (99.7%, AR grade), acetone (99.5%, AR grade), 1-methyl-2-pyrrolidinone (NMP, 99%, AR grade), triethylamine (TEA, 99%, AR grade), and methanol (99.5%, AR grade) bought from Aladdin (Shanghai, China) were employed as received, without any treatment.

Poly(vinylidene fluoride-chlorotrifluoroethylene) with inner double bonds (P(VDF-CTFE-DB), with VDF:CTFE:DB = 80:10:10 in molar ratio, with a density of ca. 1.2 g cm^−3^) was prepared from P(VDF-CTFE) by elimination of hydrogen chloride molecules [47]. P(VDF-CTFE) of 5.0 g was fully dissolved in 100 mL of NMP into a 250 mL double-necked flask containing condenser and magnetic stirrer. TEA of 30 mL (214 mmol) was added into the flask and the conversion process was executed at 70 °C under vigorous stirring for 1 h. The as-obtained mixture was slowly precipitated in deionized water of 1 L followed by washing the precipitant completely with deionized water (4 times) and then methanol (twice). The desired polymer product was dried in a vacuum oven at 45 °C for two days.

The fabrication of the nanocomposite films was as follows: both of the nanocomposite systems, namely SiC/P(VDF-CTFE-DB) and SiC/DBP/P(VDF-CTFE-DB) composite systems, were fabricated into films by a solution cast process [51] from a suspension containing a series of volume loading contents of SiC NPs in a high-viscosity polymer solution, with acetone as solvent, at room temperature, on glass slide substrates. The reason for using a high-viscosity polymer solution was to inhibit the sedimentation of SiC NPs (with higher density than P(VDF-CTFE-DB)) during the evaporation of acetone and thus to achieve a more homogeneous dispersion of SiC NPs in the polymer matrix. The weight ratio of DBP (as a plasticizer for polymer) and polymer was fixed to be 1:100 in all of SiC/DBP/P(VDF-CTFE-DB) composite films. After a thermal treatment of 160 °C in a vacuum oven for 4 h, to remove air voids (air defects) and compact filler/polymer interface regions, those nanocomposite films (with a thickness of ca. 60 μm) were completely peeled off from the glass slides, followed by sputtering with Au on both surfaces as electrodes for subsequent electric performance tests. For an easy indication, the above P(VDF-CTFE-DB) was further simplified into PVDF in the current work.

Characterization methods were as follows. X-ray diffraction (XRD) results were obtained through a Rigaku D/max 2400 diffractometer (Rigaku Industrial Corp., Tokyo, Japan) with X-ray wavelength of 1.542 Å (Cu Kα radiation, 40 kV, 100 mA), 2*θ* diffraction angle at 30–75°, rate of 15°/min and step of 0.02°. Field-emission scanning electron microscopy (FE-SEM) images were acquired using a JEOL JSM-6700F (Japan Electron Optics Laboratory Co., Ltd., Tokyo, Japan) at 15 kV. The particle size distribution histogram was obtained based on a Winner2000M laser particle size analyzer (Jinan Winner Particle Technology Co., Ltd., Jinan, China). Proton nuclear magnetic resonance (^1^H NMR) curves were obtained by a Bruker (Advance III) 400 MHz spectrometer (Bruker Corp., Karlsruhe, Germany) with acetone-d6 as solvent and tetramethylsilane as internal standard. Stress-strain relations were gained by a material testing machine CMT 6503 (Shenzhen Suns Technology Stock Co., Ltd., Shenzhen, China). Note that the tensile speed of samples was fixed at 2 mm min^−1^. Before measurements, the films were cut into dumbbell-shaped samples in which the measuring parts should have a width of 4 mm and a thickness of ca. 0.14 mm. Electric breakdown strengths were achieved by an auto voltage withstanding tester (RK2674B, Shanghai Shuangxu Electronics Co., Ltd., Shanghai, China). Dielectric and alternative current (AC) conducting performances at room temperature were gained on a HP4284A LCR meter (Hewlett-Packard, Palo Alto, CA, USA) under a testing frequency of 100 Hz–1 MHz with a voltage of 1 V. Electrical conductivity data under a direct current (DC) field of 1 kV cm^−1^ were obtained using a digital megger (PC68, Shanghai Shuangxu Electronics Co., Ltd., Shanghai, China). Au electrodes were deposited on the two surfaces of film samples by a JEOL JFC-1600 auto fine coater (Japan Electron Optics Laboratory Co., Ltd., Tokyo, Japan) before all of the electrical feature tests. However, neat SiC NPs, namely the sample bearing filler of 100 vol %, should be compressed into a cylindroid mold (diameter 20 mm, height 5 mm) to determine the electric properties. Note that the mold had two Cu electrodes at its top and bottom.

The weight cup method [50] was adopted to determine the degree of compactness for the interface zones of composite samples. Firstly, the specific gravity cup was fully filled with distilled water, followed by placing it into a thermostatic water bath at 20 °C for 30 min. After that, the cup was taken out and its exospore was dried. The weight of the cup filled with water was measured to be *W*_1_. In the end, the average density of the entire sample could be calculated. Specific steps were as follows. While preparing the sample, the total mass of SiC NPs, PVDF and DBP introduced was known and was expressed as *W*_2_. The entire sample with a weighed mass of *W*_3_ (including the mass of air inside the sample) was placed into the empty and dry specific gravity cup and the distilled water was further introduced to fully fill the cup. The bubbles in the cup as well as on the surface of the sample were removed. After a thermostatic water bath at 20 °C for 30 min, the external surface of the cup was fully dried, followed by weighing it as *W*_4_. The average density of the sample (*ρ_sample_*) and the mass of all the air inside the sample (*W_air_*) could be calculated based on Equations (1) and (2), respectively:

(1) $$\rho_{sample}=\frac{W_{3}\rho_{water}}{W_{3}+W_{1}-W_{4}}$$

(2) $$W_{air}=W_{3}-W_{2}$$

The total volume of air inside the sample (*V_air_*) is equal to (*W_air_*/*ρ_air_*). Note that both *ρ_water_* and *ρ_air_* are known. The overall volume of the sample (*V_sample_*) is (*W*_3_/*ρ_sample_*). Therefore, the volume content of air voids in the entire sample (*φ_air_*) is equal to (*V_air_*/*V_sample_*). Based on the definition, the degree of compactness is equal to 1/*φ_air_*. To realize higher readability, the abbreviations of all the long names are shown in Table 1.

## 3. Results and Discussion

### 3.1. Characterization of SiC NPs and Polymers

**Figure 1:**
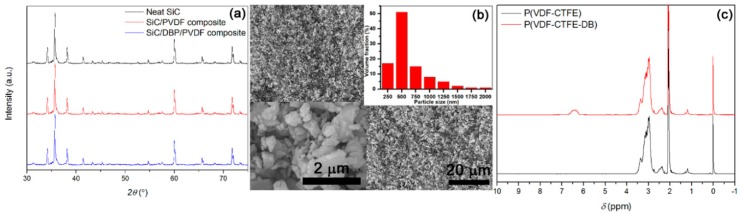
(**a**) XRD confirms SiC is alpha phase and the content of SiC in the composites is 36 vol %; (**b**) SEM shows that particle sizes range from 250 to 2000 nm, with average diameter of 500 nm, and the particles are irregular, as well as distribution histogram of particle size confirms average diameter as 500 nm; and (**c**) ^1^H NMR confirms VDF/CTFE/DB = 80/10/10 mol % in P(VDF-CTFE-DB).

The composition and morphology of the SiC NPs were investigated through XRD and SEM, as shown in Figure 1a,b, respectively. In Figure 1a, the utilized SiC NPs were confirmed to belong to high-purity alpha crystalline form. That meant SiC NPs had a structure of hexagonal crystal system according to MDI Jade 5.0 analysis software (Materials Data Management, Inc., Indianapolis, IN, USA). The diffraction angles (2*θ*) at 34°, 36°, 38°, 41°, 60°, 66° and 72° can be ascribed to crystal indices of (1 0 1), (1 0 2), (1 0 3), (1 0 4), (1 1 0), (1 0 9) and (2 0 2) in alpha-SiC NPs, respectively [52]. Neither PVDF nor DBP blended with SiC could result in the change of crystalline form of SiC based on Figure 1a. In Figure 1b, a relatively wide particle size distribution of NPs (250–2000 nm) was seen. The inset of Figure 1b could confirm an irregular 3D shape of the NPs, suggesting a high geometrical asymmetry of NPs. The average diameter of NPs was found to be 500 nm in terms of statistical method (see the distribution histogram of SiC particle size in the inset of Figure 1b).

The composition of P(VDF-CTFE-DB), characterized by ^1^H NMR, is shown in Figure 1c. The peaks situated at 2.2–2.7 ppm and 2.7–3.2 ppm were ascribed to head-head (–CF_2_–CH_2_–CH_2_–CF_2_–) and head-tail (–CF_2_–CH_2_–CF_2_–CH_2_–) connections of VDF structural units, respectively. The peak lying at 3.2–3.6 ppm was attributed to the protons (in VDF units) that were neighboring to CTFE structural units (–CF_2_–CH_2_–CFCl–CF_2_–). In comparison with P(VDF-CTFE) precursor, the novel multiple peaks situated at 6.2–6.7 ppm in as-prepared P(VDF-CTFE-DB) can be ascribed to the protons on inner double bonds (–CF_2_–CF=CH–CF_2_–) after the elimination of hydrogen chloride from (–CF_2_–CFCl–CH_2_–CF_2_–) [53]. Chemical composition of as-prepared P(VDF-CTFE-DB) could be confirmed to be VDF units of 80 mol %, CTFE units of 10 mol %, and DB units of 10 mol % based on area integrals of the result in Figure 1c.

### 3.2. Degree of Interface Compactness and Its Influence on Mechanical Properties

**Figure 2:**
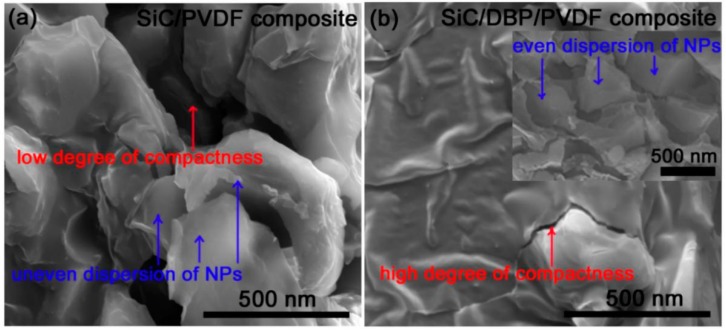
Cross-section SEM results confirm (**a**) the low compactness degree in interface zones of binary composite and (**b**) the high compactness degree in interface zones of ternary composite; the inset shows the even distribution of NPs, and both composites are filled with 36 vol % SiC.

Overload of NPs with a rather large specific surface area and low cohesive force between NPs and the polymer matrix will lead to air voids and a low degree of compactness in the interface regions [54]. In the present work, introducing a small molecule plasticizer (acting on polymeric matrix) instead of a surface-modifying NPs was adopted to elevate the degree of compactness of the interface zones [55,56,57]. As shown in the cross-section morphology of SiC/PVDF composite bearing NPs of 36 vol % in Figure 2a, air voids and a low interface adhesion could be observed. That suggested a low degree of compactness of the interface zones in the SiC/PVDF composite system. In addition to the undesired air voids introduced at interface zones, an inhomogeneous scattering of SiC NPs in the polymer matrix was observed as well, which could suggest the agglomeration of NPs during the formation and thermal treatment of composite film. Because of the low adhesion of PVDF onto the surfaces of NPs as well as the high cohesion among PVDF macromolecules, it was difficult to remove air voids from the composite, although the composite film was treated at a high temperature of 160 °C.

However, once DBP at low concentration (1 wt % of PVDF) was incorporated into the SiC/PVDF composite system, the formation of air voids in interface regions became weak in the ternary composite film (bearing NPs of 36 vol % as well), as displayed in Figure 2b, in comparison with the binary composite film in Figure 2a. The DBP raised the degree of compactness for the filler/matrix interface regions based on the weight cup method mentioned above. For an instance, the air volume fraction and the compactness degree for the 36 vol % NPs filled ternary composite were 1.2 vol % and ca. 83, respectively, while both of them for the corresponding binary composite were 4.8 vol % and ca. 21, respectively. That suggested that most of the air voids in the interface zones could be extruded from the ternary composite based on the high plasticization of PVDF matrix during the fabrication of composite film, especially during high-temperature treatment. Furthermore, the unfunctionalized SiC NPs were found to be evenly scattered in PVDF (see the inset of Figure 2b) with the help of DBP, which might be attributed to a significant decline of cohesion among PVDF macromolecules that were well isolated by DBP molecules (hydrogen bonds might be formed between the ester groups from DBP and VDF units from PVDF [58,59,60]). The elevated motion capacity of PVDF molecule chains due to a combination of DBP and high temperature might be responsible for the strong extrusion of interface air voids and the compelled close contact of neat SiC NPs and PVDF matrix. In summary, DBP might reduce the cohesion of PVDF material and thus a PVDF material with an enhanced flowability might effectively disperse the SiC NPs, restrain the uniting of SiC NPs, eliminate the air voids, and improve the interface compactness degree.

**Figure 3:**
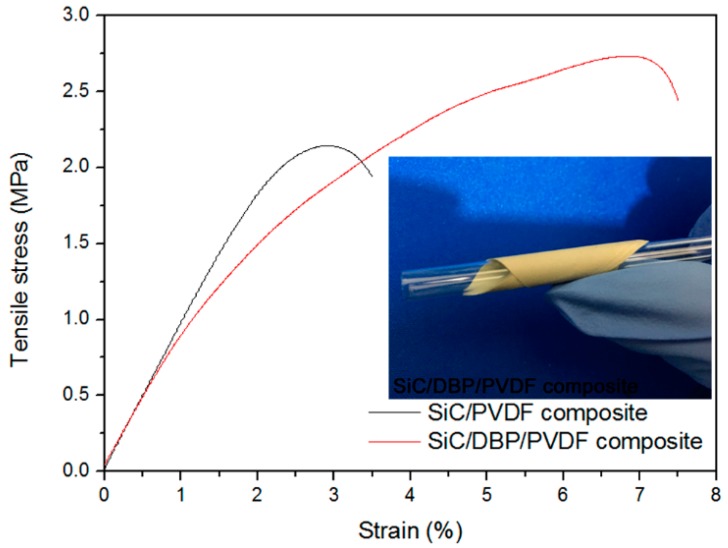
Stress-strain curves show higher mechanical property for the ternary composite compared with the binary composite, and NPs contents are both 36 vol %; the photograph shows the high flexibility for that ternary composite.

Apart from the significant impact on the dispersion of NPs in matrix, the degree of compactness of interface zones is closely connected to the improved mechanical performance of composites as well. Mechanical properties such as breaking strength, flexibility, and breaking elongation decrease once high loadings of NPs are added into polymeric materials [61]. As expected, the breaking strength and elongation of SiC/PVDF composite bearing NPs of 36 vol % were measured to be relatively low, as shown in Figure 3. The elongation and strength at break of this composite film were found to be (3.00 ± 0.36)% and (2.20 ± 0.24) MPa, respectively. That meant a poor mechanical property was obtained in the present binary composite film, which could stem from the uneven dispersion of NPs in matrix and the low interface compactness degree observed in Figure 2a. However, once the DBP in a very low loading content was introduced to construct the ternary SiC/DBP/PVDF composite filled with NPs of 36 vol %, both the measured breaking strength and the elongation of composite film were elevated in comparison to the binary counterpart. In Figure 3, the strength and elongation at break of the ternary composite film were detected to be (2.80 ± 0.31) MPa and (7.00 ± 0.84)% respectively, which could be ascribed to the homogeneous dispersion of NPs and the high interface compactness extent shown in Figure 2b. Moreover, the ternary composite film with NPs of 36 vol % was found to possess a high flexibility, as indicated in the inset of Figure 3. Last but not least, different from the dominant elastic deformation (linearity) of the binary composite before the failure, the dominant plastic deformation (non-linearity) of the ternary composite before the failure could be observed based on Figure 3. That suggested the different failure mechanisms between both composites, which might be clarified by the DBP-induced decrease in the cohesion of the polymer matrix in the ternary composite (in comparison with the binary composite). In the binary composite, a high cohesion of high polarity polymer matrix would lead to a strong capacity of the elastic deformation in polymer matrix before the failure. However, in the ternary composite, DBP-induced increase of the flowing capacity of the polymer matrix would result in the plastic deformation of the polymer matrix instead of the elastic deformation before the failure. 

### 3.3. Electric Breakdown Strength of Nanocomposites

**Figure 4:**
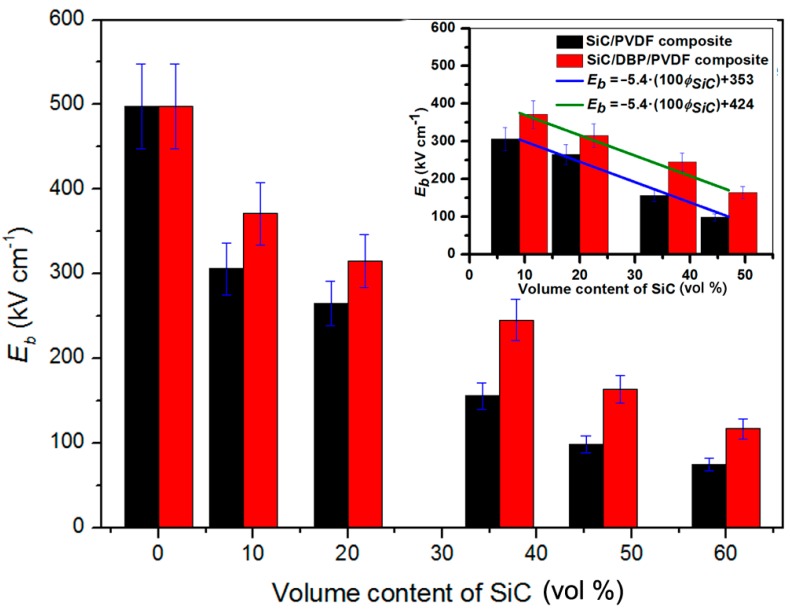
Breakdown strengths show the higher values for the ternary composites than for the corresponding binary composites, with the increase of SiC volume content; the inset shows the slope of two fitted straight lines and the difference between the intercepts of both composite systems from the DBP plasticizer.

In most cases, the electric breakdown strength (*E_b_*) of polymer-based nanocomposites is gradually reduced as the loading content of NPs is elevated. Based on the significant effect of electric breakdown strength on energy storage properties [62], endowing nanocomposites with as high an electric breakdown strength as possible is essential for researchers to obtain a sufficiently high energy storage density in resultant nanocomposites. As shown in Figure 4, the measured *E_b_* results of binary composites as a function of the volume content (0–60 vol %) of SiC NPs were given. While the NPs content increased, the *E_b_* of composites was found to decrease. That might be caused by the inhomogeneous electric field distribution across the total composites. That uneven field distribution might stem from the discrepancy of electric features of SiC and PVDF, the uniting of SiC NPs, and the loose interface zones (see Figure 2a). However, if DBP was added, the ternary composites could show higher *E_b_* than the binary counterparts. The reduced content of interface air voids and the elevated homogeneity of field distribution from the plasticizer inducing high, even dispersion of NPs and dense interface regions (see Figure 2b) could be responsible for the higher *E_b_* achieved in ternary composites. Even though SiC NPs of as high as 36 vol % were employed, the ternary composite could still exhibit an *E_b_* half that of neat PVDF material. Based on the inset of Figure 4, the *E_b_* of both composite systems was linearly fitted when the volume contents of NPs varied from 9 vol % to 47 vol %. The same slope (−5.4) for both fitted straight lines was found, suggesting a close connection between *E_b_* reduction and SiC loading. Moreover, the offset for *E_b_* between both of the composite systems was calculated to be 71 kV cm^−1^, which could be ascribed to the introduced DBP plasticizer. That is to say, adding only a small amount (1 wt % of polymer matrix) of DBP plasticizer is able to raise the *E_b_* of the ternary composites by as much as 71 kV cm^−1^ compared with the corresponding binary composites. 

### 3.4. Dielectric and Conductive Traits of Ternary Nanocomposites

**Figure 5:**
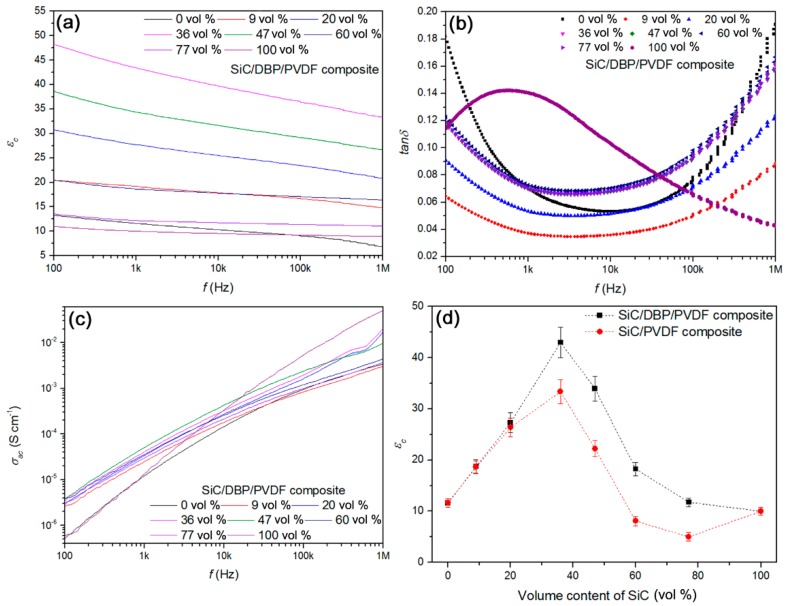
(**a**) Increase of dielectric constant for the ternary composites via blend strategy; (**b**) lower low-frequency loss for the ternary composites compared with PVDF matrix; (**c**) AC conductivity results show the fine insulating property for all of the ternary composites; and (**d**) dielectric constant at 1 kHz shows “^” trend for the binary and ternary composites with the increase of SiC volume content.

Dielectric constant (*ε_c_*), dielectric loss (*tanδ*) and ac conductivity (*σ_ac_*) of the ternary composites as a function of testing frequency (*f*) were achieved as displayed in Figure 5a–c, respectively. Based on Figure 5a, in the overall measuring frequency scope, the dielectric constant of PVDF decreased from 13 to 7 and that of SiC NPs slowly reduced from 11 to 9. Once NPs, PVDF and DBP were blended, the *ε_c_* was found to show a decreasing trend with increasing the testing frequency. That trend was similar to that of PVDF or NPs. While the loading content of NPs increased from 0 vol % to 36 vol %, the *ε_c_* gradually increased. However, as the content of NPs increased from 36 vol % to 100 vol %, the *ε_c_* rapidly reduced. Interestingly, higher *ε_c_* obtained could result in the quicker decreasing rate of *ε_c_* itself with the increase of frequency. That suggested that the elevated high *ε_c_* could be closely dependent on the lower frequency as well as the interface interaction [63]. All of the ternary composites could exhibit a higher dielectric constant than that of PVDF and SiC NPs, which would be shown later.

According to Figure 5b, the *tanδ* of neat PVDF was observed to have a changing trend of “U” shape as the f varied from 100 Hz to 1 MHz. The high *tanδ* at 100 Hz–1 kHz could be ascribed to the ionic leakage conductance across the amorphous PVDF film [53] with lower mechanical modulus. Moreover, high *tanδ* at 100 kHz–1 MHz is caused by the orientation relaxation of dipoles in high polarity VDF units. *Tanδ* of the ternary composite bearing NPs of 9 vol % is reduced in the lower frequency range in comparison to that of neat PVDF. That might stem from the decline of ionic conduction from the promotion of the overall modulus when the hard SiC NPs were introduced. Further increasing of NPs content could raise the *tanδ* of composites at lower frequency, based on improving the content of interface regions. Additionally, the *σ_ac_* (in logarithmic steps) of composites increased in a quasi-linear style as the *f* (in logarithmic steps as well) was increased from 100 Hz to 1 MHz (see Figure 5c). That could indicate their dielectric property rather than conductive one under a lower applied field (1 V). The rather close *σ_ac_* results in those composites except neat PVDF and SiC at a lower *f* could suggest the restricted low interface leakage conductance in those composites, even though the semi-conducting SiC NPs of up to 77 vol % were incorporated.

As discussed above, the incorporation of DBP plasticizer into SiC/PVDF composite system could improve the degree of compactness of interface zones and the dispersion of NPs in PVDF. The specific impact of the promoted interface compactness degree on dielectric properties of SiC/PVDF composites was researched through side-by-side comparing dielectric features of composites containing DBP and bearing no DBP. The *ε_c_* results (at 1 kHz) of composites with DBP and without DBP as a function of NPs volume content were shown in Figure 5d, respectively.

When the loading content of SiC NPs was increased, the *ε_c_* for the two composite systems showed a similar “^” shape. That is to say, *ε_c_* increased, reaching the highest value at NPs content of 36 vol %, followed by reduction. Strictly speaking, there were two datum points deviating from the “^” shape changing trend, namely *ε_c_* results of the binary composites with NPs of 60 vol % and 77 vol %. That might be ascribed to the overload of air voids in both of the composites. In both cases, the permittivity of the two composites was even lower than that of neat PVDF and SiC NPs. More importantly, the *ε_c_* results of the ternary composites were found to be much higher than that of the binary counterparts at the same volume content of NPs, which was more pronounced at NPs contents of over 20 vol %.

The dielectric constant (as a variable) of SiC has been found to be decided by the degree of interface interaction between polymer and SiC [47], and that interaction degree is dependent on the interface compatibility, the polarity of polymer materials, the grain size of SiC, the degree of polymer coating onto SiC, the loading content of SiC and the content of interface air voids [47,64]. Based on Figure 5d, while the content of SiC NPs increased from 0 vol % to 36 vol %, the nearly linear increasing trend of *ε_c_* (especially in the ternary composites) was observed, which could be attributed to the almost linear increase in the overall interface area and interaction degree between SiC NPs and PVDF. The deviation of linearity for the 36 vol % NPs filled binary composite might be ascribed to the decrease of interface compactness degree (ca. 21) in comparison with that (ca. 83) for the 36 vol % NPs filled ternary composite. Once the NPs of 36 vol % were introduced, the largest interface area and the strongest interface interaction between SiC and PVDF could be incidentally achieved. That could result in the maximum *ε_c_* value.

**Figure 6:**
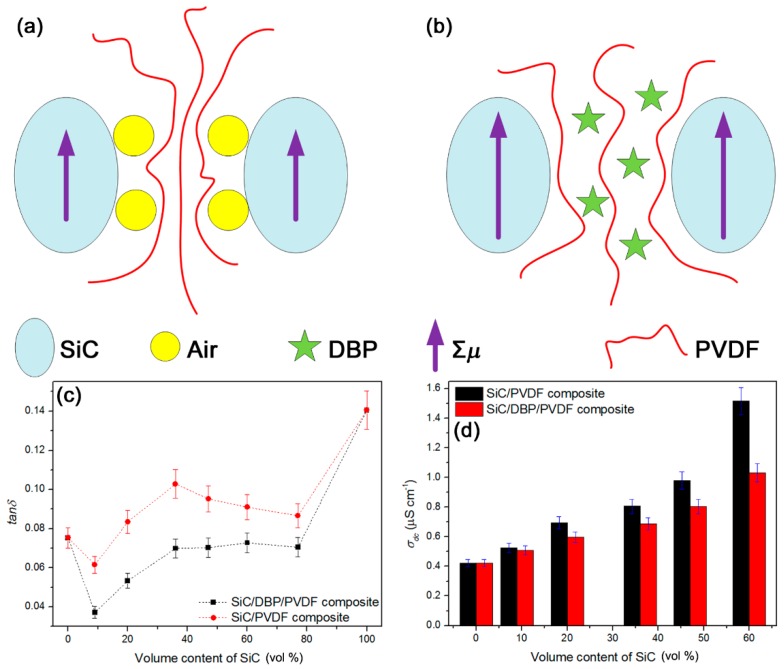
(**a**) Diagram model shows a low compact degree for the interface zones of the binary composites; (**b**) diagram model shows a high compact degree for the ternary composites; (**c**) the reduction of dielectric loss at 1 kHz for the ternary composites compared with the binary composites; and (**d**) the reduction of dc conductivity at 1 kV cm^−1^ for the ternary composites compared with the binary composites.

However, if the content of SiC NPs was further increased from 36 vol % to 77 vol %, *ε_c_* was found to reduce, instead of increasing linearly. That should stem from the decreased composition-dependent dielectric constant of SiC NPs from the elevating of the content of interface air voids (see Figure 6a) that were introduced along with the overload of NPs [47]. In other words, the degree of interface-induced polarization [47] was reduced due to the decreased degree of the compactness (from 83 to 8 for the ternary composites with the SiC content increasing from 36 vol % to 77 vol %) of SiC/PVDF interface regions. When the content of SiC NPs increased above 36 vol %, the encapsulating ratio of PVDF matrix onto all of introduced NPs would be reduced. In this case, the content of interface air avoids would be increased. The air-voids-induced new interfaces (air/PVDF and air/SiC interfaces) would continually replace the original SiC/PVDF interface, leading to the decline in *ε_c_* observed (only the SiC/PVDF interface could contribute to the increase in *ε_c_*). Combining the results in Figure 2 and Figure 5d suggests that the air voids in interface zones could be detrimental to the increase of *ε_c_* from SiC/PVDF interface-induced polarization. In summary, the high compactness degree of interface regions could give rise to the high *ε_c_* in the present composite system, and the introduction of small molecule plasticizers in a low concentration could improve the *ε_c_* of composites by extruding the interface air voids.

In Figure 6a, we show a model of the binary composite system. Due to the high cohesion among PVDF macromolecules with high polarity, as well as the poor compatibility between organic PVDF and inorganic SiC, air voids were inevitable in the SiC/PVDF interface zones at a high loading content of NPs. The air voids induced a reduction of SiC/PVDF interface interaction and explain the lower *ε_c_* of binary composites compared to the ternary ones observed in Figure 5d. The purple arrow inside the single SiC NP indicates the overall induced electric dipole moment (Σ*μ*) in SiC NP from the interface-induced polarization [65] between SiC and PVDF. By comparison, a model of the micro-structure of the ternary composite system is shown in Figure 6b. Because of the strong plastification of DBP towards PVDF matrix, the cohesion of PVDF could be reduced. That could result in a high short-range motion capacity of PVDF, especially in the high-temperature treatment. With the help of DBP molecules, PVDF molecules were driven to the surfaces of SiC NPs and thereby the air voids previously situated in the SiC/PVDF interface zones were removed from the composites. In the present case, the compact interface regions would contribute to the strengthened induced polarization between SiC NP and PVDF, leading to the larger Σ*μ* depicted in Figure 6b. That could further explain the higher *ε_c_* of ternary composites in Figure 5d.

Besides the notable effect on *ε_c_* of composites, the increased degree of compactness of the SiC/PVDF interface regions facilitated the reduction of *tanδ* and direct current conductivity (*σ_dc_*) of the composites. The *tanδ* (at 1 kHz) and *σ_dc_* (at 1 kV cm^−1^) results of those ternary and binary composites as a function of volume fraction of SiC NPs are shown in Figure 6c,d, respectively. In Figure 6c, the *tanδ* of those ternary composites bearing NPs of 9–77 vol % was found to be always lower than that of neat PVDF. That suggested the *tanδ* of those composites from the interface-induced polarization was restrained by introducing hard SiC NPs to promote the modulus of composites and depress the ionic leakage conduction. The *tanδ* of those binary composites showed a similar changing trend to that of the above ternary composites. However, the *tanδ* of binary composites was higher than that of ternary counterparts, which indicates a larger leakage-conduction-induced *tanδ* from the inhomogeneous dispersion of NPs and the low compactness degree of interface zones. According to Figure 6d, the *σ_dc_* of ternary composites was lower than that of binary composites in a wide NPs volume content range, which might be attributed to the increase in the compactness degree of interface zones as well as the elevated dispersion of NPs in PVDF. To sum up, the desired high *ε_c_*, low *tanδ*, and low *σ_dc_* could be simultaneously achieved in the present SiC/DBP/PVDF ternary composites based on high-temperature plastification of polymers.

## 4. Conclusions

- Instead of introducing a surface modification to the filler, an organic plasticizer was added to soften the polymer matrix for fabricating polymer-based nanocomposites.
- After incorporating the plasticizer, SiC/DBP/PVDF composites had a stronger dispersion of SiC, fewer air voids, and a higher compactness degree than SiC/PVDF composites.
- Adding the plasticizer led to an increase in the dielectric constant, breakdown strength, tensile properties, and flexibility, as well as a reduction of loss and conductivity.
- High dielectric and mechanical properties were balanced for the composites via combining high temperature with organic plasticizer.

## Figures and Tables

**Table 1:** Abbreviations for all the long names to increase the readability.

Original Names	Abbreviations	Original Names	Abbreviations
poly(vinylidene fluoride-chlorotrifluoroethylene) with inner double bonds	P(VDF-CTFE-DB); PVDF	nanoparticles	NPs
dibutyl phthalate	DBP	high-dielectric-constant	High-k
poly(vinylidene fluoride-chlorotrifluoroethylene)	P(VDF-CTFE)	1-methyl-2-pyrrolidinone	NMP
triethylamine	TEA	X-ray diffraction	XRD
field-emission scanning electron microscopy	FE-SEM	proton nuclear magnetic resonance	^1^H NMR
alternative current	AC	direct current	DC
weight of cup filled with water	*W*_1_	total mass of SiC, PVDF and DBP introduced	*W*_2_
weighed mass of entire sample	*W*_3_	weight of cup, sample and water	*W*_4_
average density of sample	*ρ* *_sample_*	mass of all air inside sample	*W_air_*
density of water	*ρ* *_water_*	total volume of air inside sample	*V_air_*
density of air	*ρ* *_air_*	overall volume of sample	*V_sample_*
volume content of air voids in entire sample	*φ* *_air_*	degree of compactness	1/*φ_air_*
diffraction angle	2*θ*	breakdown strength	*E_b_*
dielectric constant	*ε* *_c_*	dielectric loss	*tanδ*
ac conductivity	*σ* *_ac_*	testing frequency	*f*
overall induced dipole moment	Σ*μ*	direct current conductivity	*σ* *_dc_*
